# Quantification of Microstructural Features and Prediction of Mechanical Properties of a Dual-Phase Ti-6Al-4V Alloy

**DOI:** 10.3390/ma9080628

**Published:** 2016-07-28

**Authors:** Dong Yang, Zhanqiang Liu

**Affiliations:** 1 School of Mechanical Engineering, Shandong University, Jinan 250061, China; me_yangdong@yeah.net; 2 Key Laboratory of High Efficiency and Clean Mechanical Manufacture, Ministry of Education, Shandong University, Jinan 250061, China

**Keywords:** microstructure quantification, mechanical property optimization, Ti-6Al-4V, milling

## Abstract

Ti-6Al-4V titanium alloy milling has been frequently used in aviation/aerospace industries. Application environments put forward high requirements to create a desired proportion of the constituent phases and fine grain size for optimum mechanical properties of the machined workpiece. However, quantifying microstructural features of dual-phase (α + β) Ti-6Al-4V titanium alloy is difficult due to its irregular geometry and large dimension span. In this paper, a novel scanning electron microscope (SEM) image processing method was proposed to identify the content of constituent phases of materials. The new approach is based on the fact that the constituent phases of Ti-6Al-4V titanium alloy show different gray levels in digital images. On the basis of the processed image, distribution and average values of grain sizes were calculated directly using Image-Pro Plus software. By the proposed method, sensitivity of microstructural changes to milling parameters is analyzed and the stress-strain behavior for two ductile phase alloys is developed. Main conclusions are drawn that Ti-6Al-4V titanium alloy milling induces a high content of β phase and small grain size on the machined surface. The maximum measured values of change rate of β phase, grain refinement rate at the machined surface, and thickness of the deformation layer are 141.1%, 47.2%, and 12.3 μm, respectively. Thickness of the deformed layer and grain refinement rate decreased distinctly with the increase of cutting speed, but increased with the increase of the feed rate. The parameter of the depth of cut played a positive role in increasing the thickness of the deformed layer, while opposite to the grain refinement rate. For the variation of the change rate of the β phase at the machined surface, depth of cut is the foremost factor among the three studied parameters. Values of yield strength varied from 889–921 MPa with the change of content of the β phase from 30%–45%.

## 1. Introduction

Milling of Ti-6Al-4V titanium alloy is the primary operating process for aviation/aerospace manufacturing industry. However, Ti-6Al-4V is known to be difficult to machine for its high chemical reactivity and low thermal conductivity, which gives rise to short tool life and poor machining quality [1]. Surface integrity, with particular regard to microstructure, determines the mechanical properties and performance of the product achieved by final machining [2,3]. Fan [4] investigated the role of microstructure on fatigue properties of Ti-6Al-4V. The result was drawn that the fatigue strength ranking from high to low is as follows: equiaxed, bimodal, Widmanstätten, and acicular α’ martensite microstructure. Chan [5] found that variation of mean stress distribution in individual microstructural units can lead to fatigue life variability in Ti-6Al-4V with a duplex microstructure. Previous studies have shown that the lamellar microstructure exhibits lower strength, lower ductility, and better fatigue propagation resistance compared with an equiaxed microstructure. Equiaxed microstructure provides better fatigue initiation resistance, but poorer propagation resistance than a lamellar microstructure. Bimodal microstructures exhibit a well-balanced fatigue properties profile, since they combine the advantages of both lamellar microstructure and equiaxed microstructures. For the different mechanical properties exhibited by various microstructural features, it is important to identify the machining-induced microstructures, and to obtain favorable microstructures.

Digital micrographs taken by scanning electron microscope (SEM) or optical metallurgical microscope are common used to identify the variation of microstructures in and beneath the machined surface. In Che-Haron’s [6] study on surface integrity of machining Ti-6Al-4V, it was found that a thin deformed layer was formed underneath the machined surface under the dry cutting condition with uncoated carbide cutting tools. A dull tool caused severe microstructure alteration when the thickness of the deformed layer was less than 10 μm. Hughes [7] showed that the thickness of the deformed layer increased with the depth of the cut, but the effect of cutting speed and feed rate was not distinct. The phenomenon of the β phase decreased at the vicinity of the machined surface was also found when dry drilling Ti-6Al-4V [8]. Different from the above findings, neither phase transformation, nor deformed layer, did Velasquez observe when high-speed dry turning Ti-6Al-4V in the high cutting speed range [9]. The main reason for these inconsistent results or doubtful conclusions is that visual inspection of microstructure micrographs is insufficient to reveal the changes in the microstructure.

To study the correlation between mechanical properties of materials and microstructural features induced by machining, quantitative microstructural information (such as grain size, which is the key parameter in Hall-Petch relation [10,11]) should be extracted from digital micrographs. Several standard test methods for determining the distribution gradient of constituent phases and average grain size have been developed [12,13]. Quantifying microstructural features in dual-phase (α + β) Ti-6Al-4V titanium alloy are still difficult. Irregular geometry and large dimension span caused by different thermo-mechanical processing methods are the two primary reasons. Alborz Shokrani [14] investigated the microstructural features in end milling of Ti-6Al-4V under dry, wet, and cryogenic conditions. Average white pixel concentration below the machined surface was identified on the basis of various image processing techniques using Matlab. Collins [15] demonstrated the possibility of developing automated or semi-automated stereological procedures to determine the average values of the grain size and the volume fraction of the constituent phases. Tiley [16] measured the grain size and volume fraction of the constituent phases using random line segments and grid of cycloids methods. Moreover, automated or semi-automated tools for image analysis of α/β Ti-alloy type microstructures were developed to determine average values of the complex microstructural features, such as the thickness of the Widmanstätten α-laths, the colony scale factor, the prior β grain factor, and the volume fraction of Widmanstätten α [17,18]. However, statistical characteristics of microstructures, such as frequency of the grain size and distribution of the volume fraction were not obtained in these studies. In the current study, a novel method of image recognition is developed to quantify microstructural features in milling Ti-6Al-4V titanium alloy. The new approach is based on the fact that the constituent phases of Ti-6Al-4V titanium alloy show vastly different gray levels in digital images. By the proposed method, the distribution gradient of constituent phases on and beneath the machined surface are identified. Meanwhile, size distribution and mean diameter of α grains were calculated using Image-Pro Plus software on the basis of the processed images. Sensitivity of microstructural changes (change rate of the constituent phases, depth of the deformation layer, and grain refinement rate) to milling parameters (cutting speed, feed rate, and depth of cut) are also investigated. On the basis of the recognized microstructural features, mechanical properties of the machined surface are controlled.

## 2. Experimental Procedure

### 2.1. Materials

**Figure 1:**
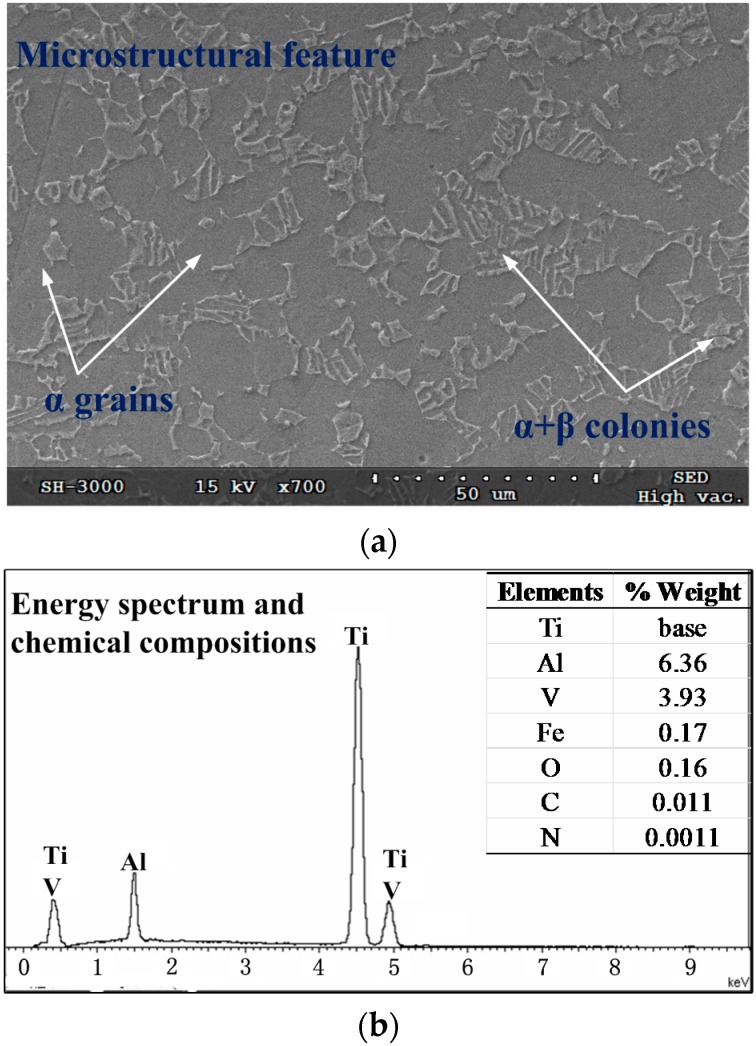
(**a**) Microstructural feature; and (**b**) energy spectrum and chemical compositions of the Ti-6Al-4V alloy studied.

The workpiece material studied was α + β two-phase Ti-6Al-4V titanium alloy, which was formed by the free forging process. Microstructural features photographed by scanning electron microscope (SEM), energy spectrum, and chemical compositions identified by energy dispersive spectroscopy (EDS) are shown in Figure 1. As shown in Figure 1, the original microstructures of the material, primary α grains shown in dark gray and lamellar α + β colonies shown in bright gray, are included.

### 2.2. Cutting Experiments

**Figure 2:**
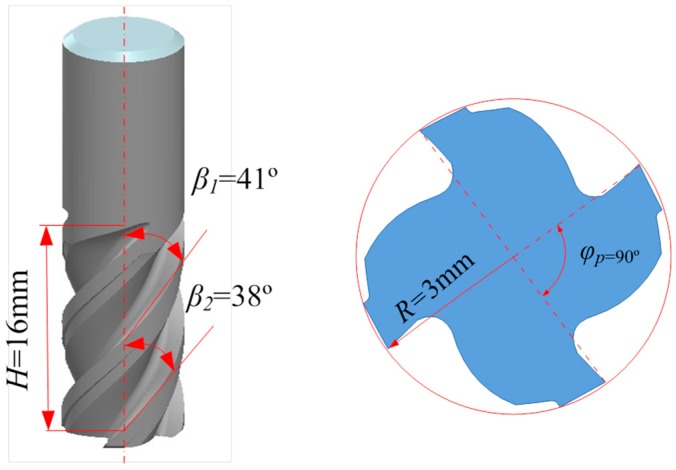
Sketch of the milling tool with 4-flute and variable helix angles.

The machining experiments were carried out on a vertical-type machining center (DAEWOOACE-V500). The workpiece dimensions were 50 mm × 30 mm × 5 mm. The cutting length was set to 50 mm. Materials for the milling cutter with four-flute and variable helix angles (38° and 41°) were cemented carbides. The diameter of the milling cutter was 6 mm. A sketch of the cutting tool is shown in Figure 2. The operation mode was down-milling. Each test sample was machined with a new tool. The aim is to assess the influence of cutting conditions on the microstructure change independently of tool wear. Machining without the use of any cutting fluid (dry or green machining) is becoming increasingly more popular due to concerns regarding the safety of the environment. Most industries apply cutting fluids/coolants when their use is not necessary. The coolants and lubricants used for machining represents 16%–20% of the manufacturing costs, hence, the extravagant use of these fluids should be restricted [19]. Based on the above considerations, the tests were performed with dry machining.

Taguchi’s L16 (4^3^) orthogonal experimental design method was applied to investigate the response of the microstructure change to cutting parameters. By reference to the cutting parameters of a certain type of aero engine casing, levels of experimental factors are selected as shown in Table 1.

### 2.3. Samples Preparation

**Figure 3:**
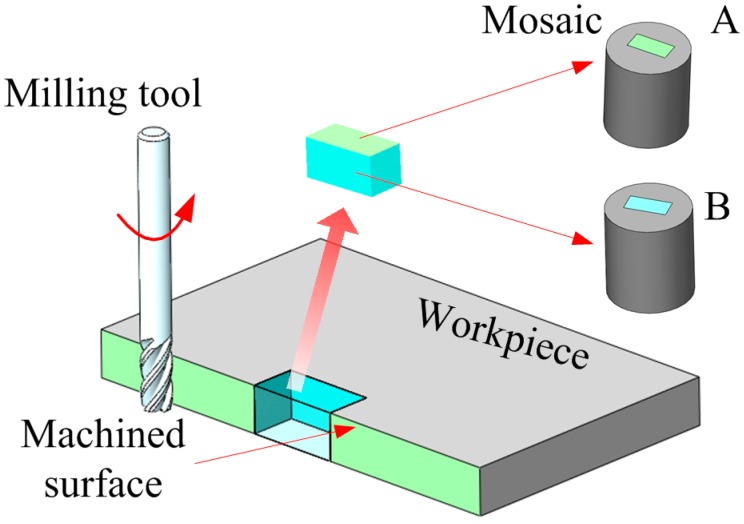
Diagram of sample preparation.

As shown in Figure 3, a slice of material was extracted from the center position of the machined workpiece. Two mosaic blocks were made to identify the microstructural features on the machined surface and the cross section. Samples were polished, and then etched in 5 mL HNO_3_ (65% conc.) + 3 mL HF (40% conc.) + 100 mL H_2_O at room temperature for 10 s (removal rate 25 nm/s) [20]. Approximately 250 nm materials were removed. A SH-3000 Mini-SEM ( HIROX, Tokyo, Japan) was utilized to image the microstructures of the samples.

## 3. Inspection of the Microstructural Features

### 3.1. Qualitative Description

**Figure 4:**
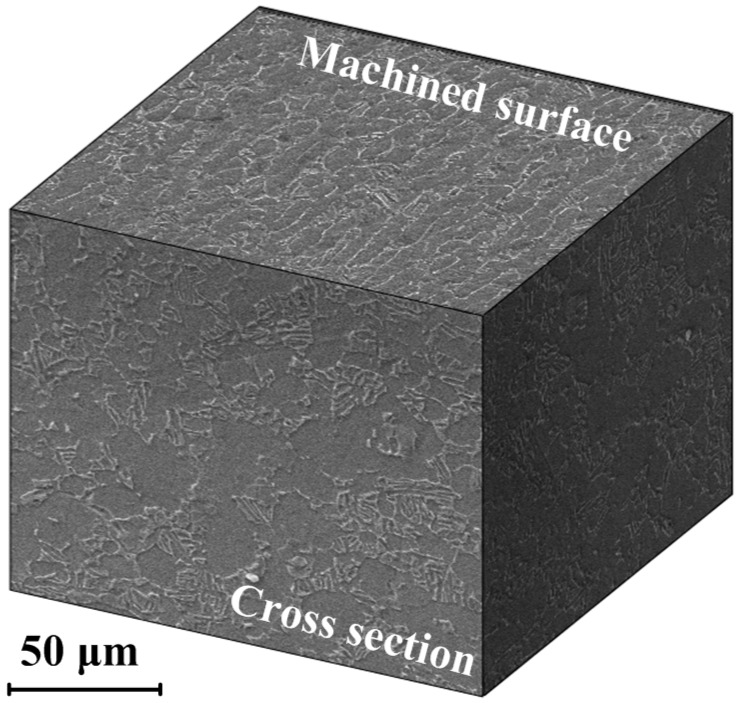
Microstructures taken from cross-section and machined surfaces of the workpiece.

Figure 4 shows the microstructures taken from the cross-section and machined surface of the workpiece at *v_c_* = 50 m/min, *f_z_* = 0.05 mm/z and *a_e_* = 2 mm. In milling operations, strain aging, and recrystallization occurred on and beneath the machined surface under the combined effect of thermal, mechanical, and chemical energy. As a consequence, plastic deformation, phase transformation, and microstructure alteration will inevitably happen. It is especially for machining Ti-6Al-4V titanium alloy, due to its high chemical reactivity and low thermal conductivity, while, in the cross-section of the sample, no remarkable microstructural alterations were observed by visual inspection. Contrary to the founding in the cross-section, obvious changes of the grain sizes and the evolution of textures were observed in the machined surface. All of the above phenomena were also found in other milling operations by visual inspection.

### 3.2. Quantitative Identification

#### 3.2.1. Proposed Method

In a SEM image of the two-phase Ti-6Al-4V titanium alloy, the constituent phases showed different gray levels, which are determined by the gray values of each image pixel. As such, contents of the constituent phases can be obtained by calculating the distribution of the gray values of image pixels. Meanwhile, some processing techniques should be applied to eliminate the influence of other factors, such as noise and uneven light.

**Figure 5:**
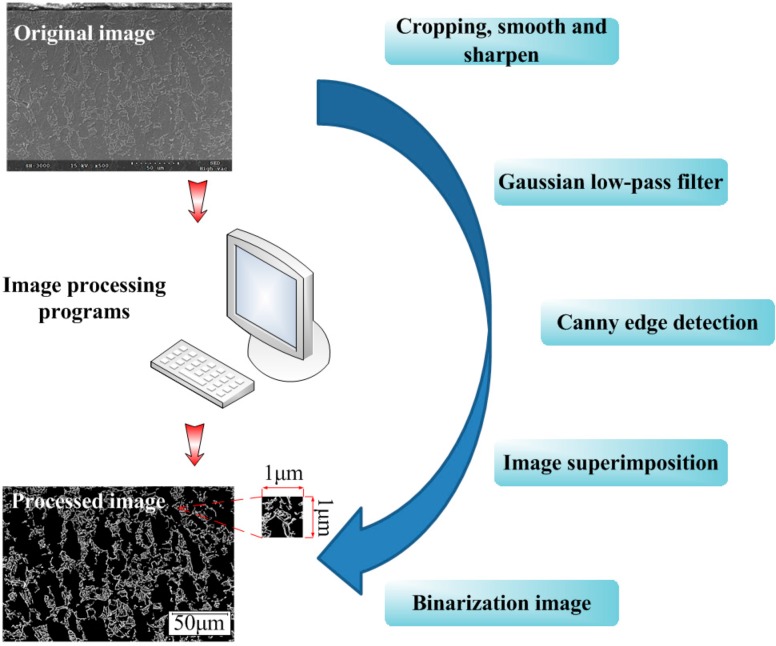
Flowchart of the image processing.

A digital image processing program was developed in Matlab. As shown in Figure 5, a flowchart of the image processing shows seven main steps, including:

(1) The original image was entered into Matlab software with an uncompressed tagged image file format. Cropping, smoothing, and sharpening were then carried out to eliminate image defects, such as uneven brightness;
(2) Noise reduction of the digital image using a Gaussian low-pass filter;
(3) Implement the Canny edge detection algorithm after executing image graying;
(4) Superposition of the images, which are processed by step (1) and step (3), to add contrast between the α and β phases;
(5) The gray image was transformed into a binary image. The image matrix of the binary image only consists of pixel values “0” and “1”, where “1” represents the α phase, and “0” represent the β phase;
(6) Extract the density of the constituent phases per square micron. The distribution gradients of the constituent phases beneath the machined surface are then obtained; and
(7) Lamellar α + β colonies were regarded as a single β phase, and then profiles of the α phase were picked up. The size distribution and mean diameter of α grains were then calculated using Image-P Plus software. The statistics cover the whole SEM image with a size of 244 μm × 167 μm. The mean diameter is the average length of diameters measured at 2 degree intervals and passing through the profile centroid of the α phase.

#### 3.2.2. Identification Results

**Figure 6:**
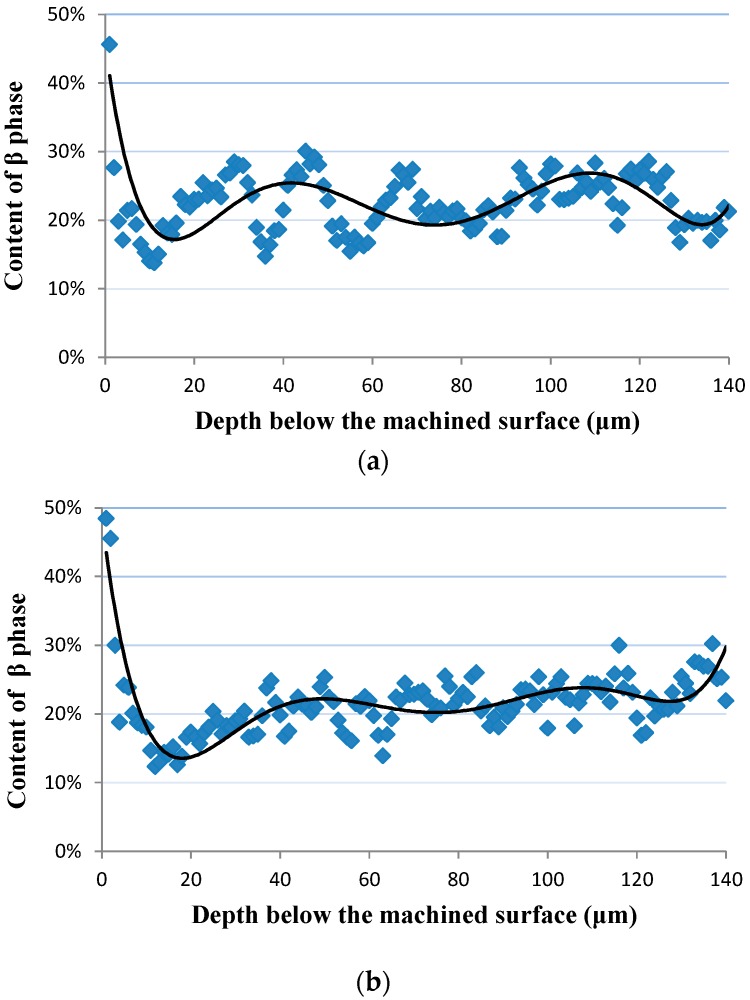
Distribution gradients of the β phase beneath the machined surface. (**a**) *v_c_* = 50 m/min, *f_z_* = 0.02 mm/z, *a_e_* = 1.5 mm; and (**b**) *v_c_* = 110 m/min, *f_z_* = 0.02 mm/z, *a_e_* = 0.5 mm.

**Figure 7:**
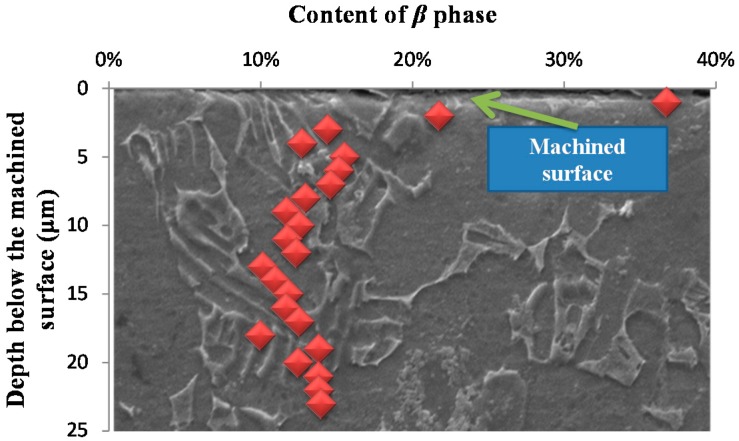
Similarity between the settle distance of the distribution gradient of the β phase and the deformed layer thickness.

Figure 6 shows the distribution gradient of the β phase beneath the machined surface. The content of the β phase (f_β_) in the matrix is ranged from 13%–30%, and the average value is 22.1%. A significant increase of the content of the β phase at the vicinity of the machined surface was observed. The distribution gradient of the β phase beneath the machined surface is consistent with micro hardness gradient beneath the machined surface. This stems from the fact that the β phase is much harder than the α phase [21]. Two key features, including the change rate of the β phase at the machined surface and the settle distance of the distribution gradient of the β phase, could be identified from the distribution gradient of the β phase. Figure 7 shows that the settle distance of the distribution gradient of the β phase can be used to reflect the deformation layer depth. According to Figure 6, values of the change rate of the β phase at the machined surface and deformation layer depth are 100.1%, 4 μm and 125.5%, 6 μm at *v_c_* = 50 m/min, *f_z_* = 0.02 mm/z, *a_e_* = 1.5 mm and *v_c_* = 110 m/min, *f_z_* = 0.02 mm/z, *a_e_* = 0.5 mm, respectively.

**Figure 8:**
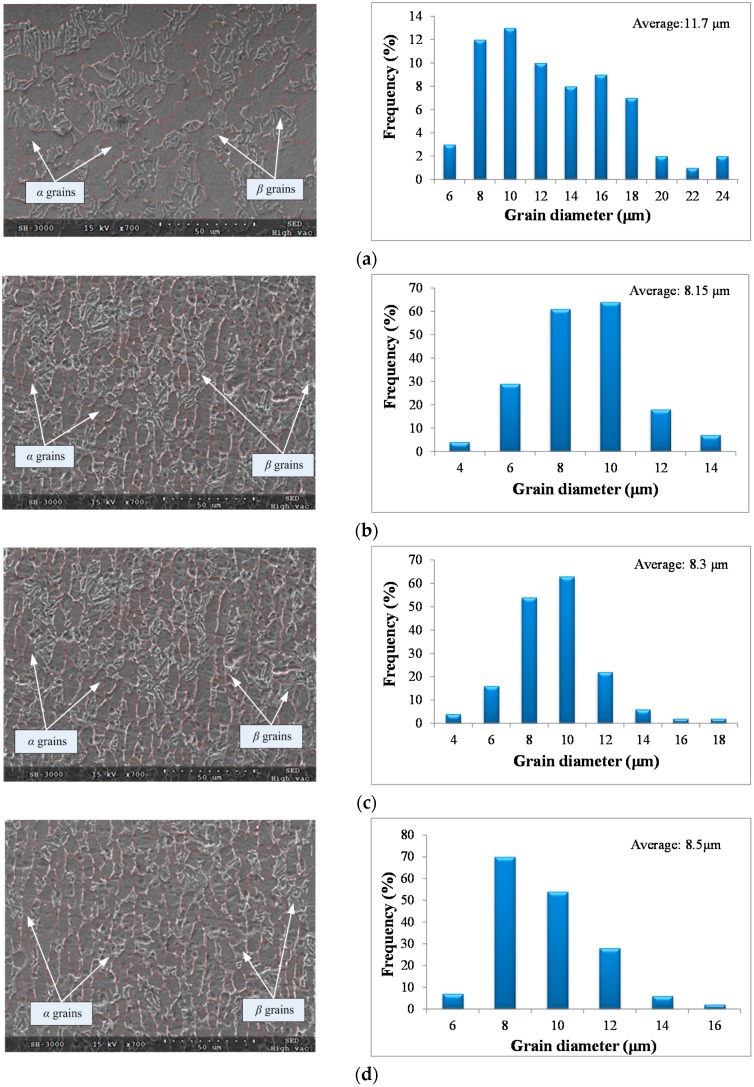
Microstructure and grain size distribution of the original material and the machined surface. (**a**) Original microstructure and frequency of grain sizes; (**b**) machined surface (*v_c_* = 20 m/min, *f_z_* = 0.03 mm/z, *a_e_* = 1.5 mm); (**c**) machined surface (*v_c_* = 50 m/min, *f_z_* = 0.05 mm/z, *a_e_* = 2.0 mm); and (**d**) machined surface (*v_c_* = 80 m/min, *f_z_* = 0.04 mm/z, *a_e_* = 1.0 mm).

Figure 8 shows the microstructures and grain size distributions of the original material and the machined surface under different cutting condition. Grain diameters of the original material ranged from 6 μm to 24 μm, and the average value is 11.7 μm. Grain refinement was noticed at the machined surface under different cutting condition. The grain refinement rates under the cutting condition of (b), (c), and (d) in Figure 7 were 30.34%, 29.1%, and 27.35%, respectively.

Under the cutting conditions of the present study, ranges of measured values of the change rate of the β phase, the grain refinement rate at the machined surface, and the thickness of the deformation layer are 46.7%–141.1%, 18.6%–47.2%, and 3.7–12.3 μm, respectively.

Microstructural parameters, such as the phase distribution gradient and the grain size frequency, are the key factors in understanding the relationship between microstructural features and mechanical properties of the machined surface. Quantification of these microstructural parameters allows cutting parameters optimization to obtain the desired mechanical properties.

### 3.3. Milling Parameter Sensitivity Analysis

Different microstructural features are generated in milling Ti-6Al-4V titanium alloy under different cutting conditions. As a consequence, the final machined workpiece will express discrepant mechanical properties. A quantitative description of the sensitivity of microstructural features to milling parameters can play a vital role in controlling the performance of the workpiece. Three main features, including the change rate of the β phase on the machined surface, the thickness of the deformed layer, and the grain refinement rate were investigated in this research.

**Figure 9:**
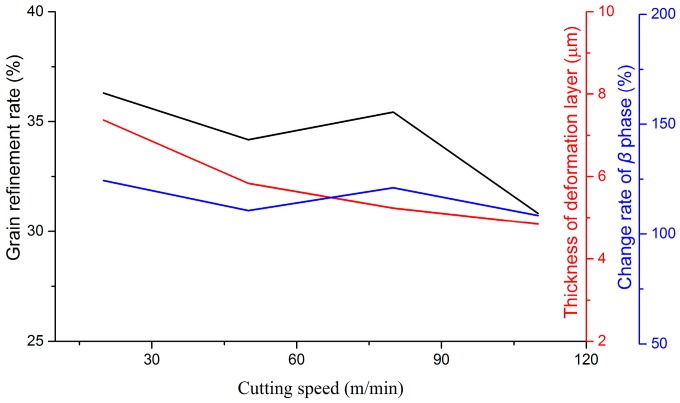
Variations of microstructural features with the cutting speed.

**Figure 10:**
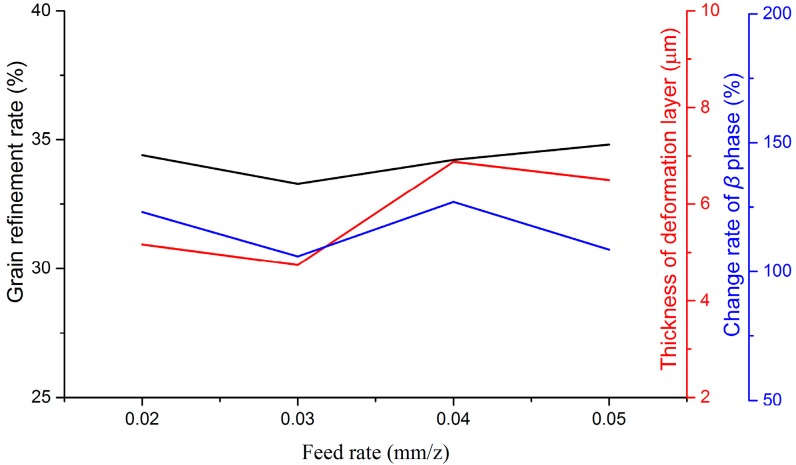
Variations of microstructural features with the feed rate.

**Figure 11:**
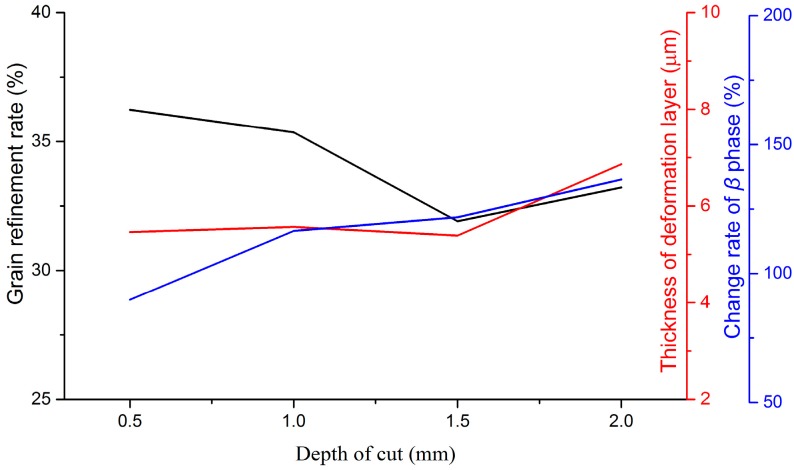
Variations of microstructural features with the depth of cut.

Variations of microstructural features with milling parameters are given in Figure 9, Figure 10 and Figure 11. Cutting speed, feed rate, and depth of cut have been approved to affect microstructural features distinctly. In the milling process, variations of microstructural features depend on various factors, such as cutting temperature, strain, and strain rate, etc. With the increase of the cutting speed, cutting temperature increased in the primary shear zone, which is in favor of recovery, recrystallization, and grain growth. On the contrary, contact duration decreased with the increase of the cutting speed. As a consequence, the deformation degree in the tool-workpiece contact zone is small. A greater cutting force and deformation can be developed by increasing the feed rate and the depth of cut. Therefore, the mechanical work and energy, which would be converted to the driving energy of the phase transformation and grain growth, went up. All of these reasons are responsible for the variation of microstructural features in milling Ti-6Al-4V titanium alloy. In the range of experimental parameters, the thickness of the deformed layer and grain refinement rate decreased distinctly with the increase of the cutting speed, but increased with the increase of the feed rate. The parameter of the depth of cut played a positive role in increasing the thickness of the deformed layer, while opposite to the grain refinement rate. For the variation of the change rate of the β phase at the machined surface, the depth of cut is the foremost factor among the three studied parameters.

## 4. Mechanical Property Optimization

Hall-Petch relation [22] expressed the dependency of the lower yield point or the fracture stress of metals on the grain size. It was also suggested for the effect of the grain size on other mechanical properties of polycrystalline metals and alloys [23]. As shown in Equation (1), the flow stress has a linear relationship with the reciprocal square root of grain size:

(1) $$\sigma_{0}=k_{1}+k_{2}d^{-0.5}$$

where $k_{1}$, $k_{2}$ are material constants, and $\sigma_{0}$ and d are flow stress and grain size.

For the reason that the Hall-Petch relation was proposed for the single phase material, originally, this relationship was not reliable for α + β two-phase Ti-6Al-4V titanium alloy. Furthermore, grain size is difficult to calculate due to the complex microstructural features of Ti-6Al-4V, which is comprised with α phase and the lamellar α + β colonies, although the Hall-Petch relation was occasionally used. For these reasons, a constituent phase content-based finite element model was proposed.

The underlying idea in modeling flow stress of the α + β two phase Ti-6Al-4V titanium alloy is to simulate a uniaxial tensile experiment. The finite element analyses were performed under constant stress increments and in the plane stress condition.

**Figure 12:**
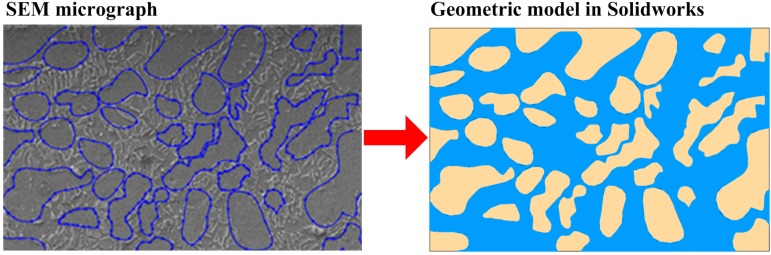
Schematic diagram of geometry modeling (f_β_ = 22.1%).

As shown in Figure 12, is the schematic diagram of geometry modeling. Micrographs of the two-phase microstructure were first obtained, where a lamellar α + β colony was regarded as a single β phase. Edges of the two phases were then mapped using Solidworks software. α phases are represented in yellow and β phases are in blue. In Solidworks/Simulation, the Ramberg–Osgood (R-O) constitutive model, shown in Equation (2), was applied. Material constants of the constituent phases are listed in Table 2.

(2) $$\varepsilon =\frac{\sigma }{E}+\frac{a^{\prime }\sigma_{y}}{E}{\left(\frac{\sigma }{\sigma_{y}}\right)}^{N}$$

where $\sigma_{y}$, *N*, a′, *E* are yield stress, inverse of the strain hardening exponent, empirical constant, and Young’s modulus, respectively. The Ramberg–Osgood constitutive equation was created on the basis of rate-independent plastic flow. Hardening behavior of the material depends on the material constants $a^{\prime }$ and *N*. Due to the power-law relationship between stress and plastic strain, the Ramberg–Osgood model implies that plastic strain is present even for very low levels of stress. Nevertheless, for low applied stresses and for the commonly used values of the material constants a′ and *N*, the plastic strain remains negligible compared to the elastic strain. On the other hand, for stress levels higher than $\sigma_{y}$, plastic strain becomes progressively larger than elastic strain.

Contact condition is set to global contact, where the constituent phases are bonded. Therefore, no contact surface separation will be produced in the process of the simulation. Moreover, incompatible mesh technology and the plane triangle element with three nodes were applied in the simulation.

**Figure 13:**
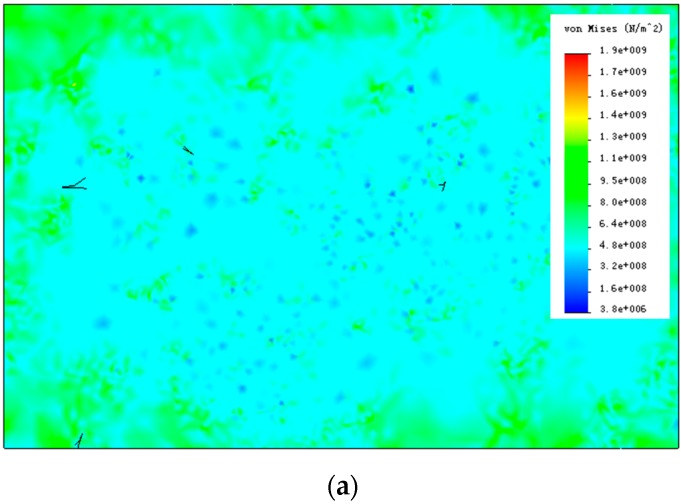

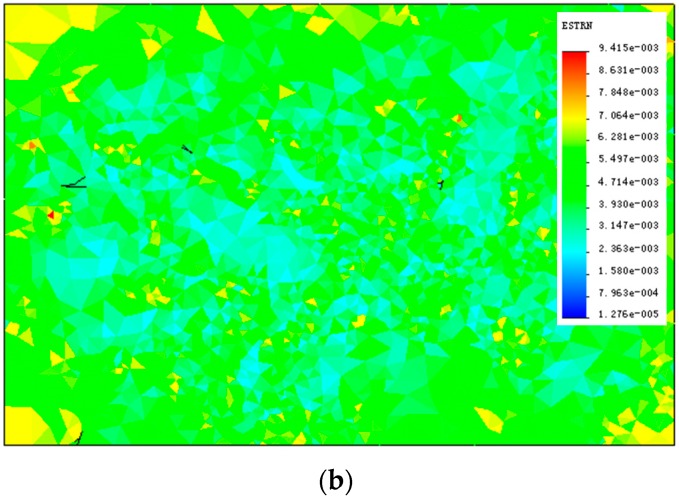
(**a**) Stress and (**b**) strain contour figures under the condition that the tensile stress is 600 Mpa (f_β_ = 22.1%).

**Figure 14:**
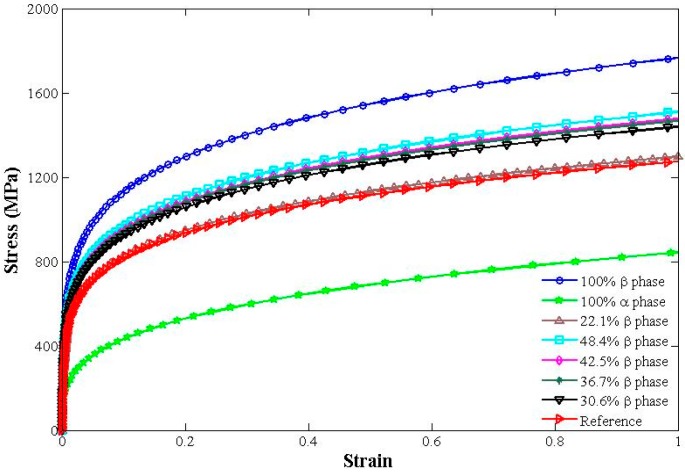
Computed stress-strain curves for various contents of β phase.

Figure 13 shows the stress and strain contour figures under the condition that the tensile stress is 600 Mpa. The average stress and strain of each state can be calculated using Equations (3) and (4):

(3) $$\bar{\sigma }=\frac{1}{V}\int_{V}^{\;}\sigma dV$$

(4) $$\bar{\varepsilon }=\frac{1}{V}\int_{V}^{\;}\varepsilon dV$$

where, $\bar{\sigma }$ and $\bar{\varepsilon }$ are the average stress and the average strain, *V* is the total volume of the microstructure model, and σ and ε are the stress computed at every Gauss point in the model. Based on the average stress and strain of each state, stress-strain curve was drawn. Seven microstructures were simulated with volume fractions of primary *a* grains ranging from 0–100%. They are all shown in Figure 14. As a contrast, a stress-strain curve (red in Figure 14) reported in [24] was chosen (strain-rate hardening and thermal softening were not considered in the present study).

The correlation coefficient (*R*), as described by Equation (5), was applied to illustrate the statistical relationship between the reference curve and the predicted one (Brown in Figure 14):

(5) $$R=\frac{\sum_{i=1}^{n}\left(x_{i}-\bar{x}\right)\left(y_{i}-\bar{y}\right)}{\sqrt{\sum_{i=1}^{n}{\left(x_{i}-\bar{x}\right)}^{2}{\left(y_{i}-\bar{y}\right)}^{2}}}$$

where *x_i_* and *y_i_* are the data point of reference and predicted curves. $\bar{x}$ and $\bar{y}$ are average values of data points of reference and predicted curves.

The calculated value of *R* was 0.893, which indicated a high degree of consistency between reference and predicted stress-strain curves. Conclusion can also be drawn from Figure 14 that a high content of β phase represented a high strength of material.

**Figure 15:**
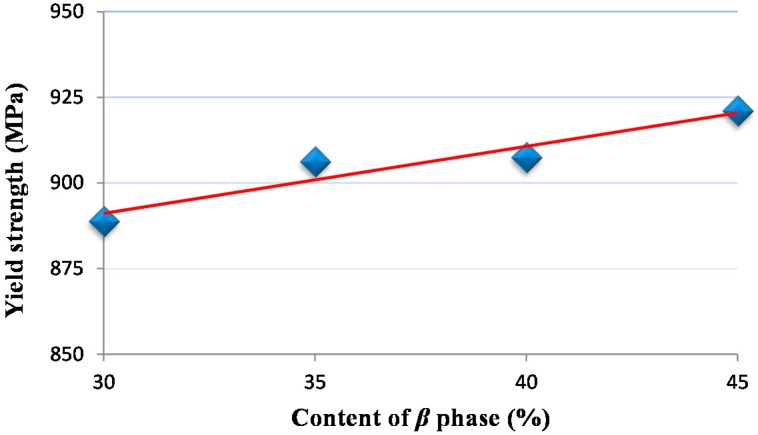
Yield strength variation of the material with content of the β phase.

Yield strength, which is measured by 0.2% offset strain method, was identified from Figure 14. According to Figure 15, values of yield strength varied from 889–921 MPa with the change of content of β phase from 30% to 45%.

The most outstanding advantage of the proposed method of characterizing microstructural features is its available in presenting gradient distribution of microstructural features beneath the machined surface. These studied microstructural features were closely related to the mechanical property as represented by the stress-strain behavior. As a consequence, the proposed method will serve as a powerful tool in developing machining parameters—mechanical property models of the dual-phase Ti-6Al-4V titanium alloy.

## 5. Conclusions

To quantify microstructural features and control the machining-induced mechanical properties in milling titanium alloy Ti-6Al-4V, a novel method of image recognition is developed to identify the microstructural changes at the vicinity of the machined surface. According to this investigation’s results, the conclusions can be summarized as follows:

1. A digital image processing method was proposed. The new approach is based on the fact that the constituent phases of Ti-6Al-4V titanium alloy show different gray levels in digital images. By the proposed method, microstructural features, including the content of constituent phases and grain size, were identified. A high content of the β phase and small grain size were found at the machined surface. The maximum measured values of change rate of β phase, grain refinement rate at the machined surface, and thickness of the deformation layer are 141.1%, 47.2%, and 12.3 μm, respectively.
2. Sensitivity of microstructural changes to milling parameters was investigated. The thickness of the deformed layer and grain refinement rate decreased distinctly with the increase of the cutting speed, but increased with the increase of the feed rate. The parameter of the depth of cut played a positive role in increasing the thickness of the deformed layer, while opposite to the grain refinement rate. For the variation of the change rate of the β phase at the machined surface, the depth of cut is the foremost factor among the three studied parameters.
3. Stress-strain behavior of two ductile phase alloys was developed using the finite element method. A high content of the β phase was found to have a high strength of materials. Values of yield strength varied from 889–921 MPa with the change of content of the β phase from 30%–45%.

## Figures and Tables

**Table 1:** Orthogonal experimental parameters.

Levels/Factors	Cutting Speed *v_c_* (m/min)	Feed Rate *f_z_* (mm/z)	Radial Depth of Cut *a_e_* (mm)
**Level 1**	20	0.02	0.5
**Level 2**	50	0.03	1.0
**Level 3**	80	0.04	1.5
**Level 4**	110	0.05	2.0

**Table 2:** Material constants of the constituent phases for Ti-6Al-4V [5].

Phase	σ_*y*_ (MPa)	*E* (GPa)	a′	*N*
**α**	345	140	17.959	3.484
**β**	1000	170	8.246	5.305
